# Case Report: Budd–Chiari-like syndrome in a cat with polycystic kidney and liver disease

**DOI:** 10.3389/fvets.2025.1701832

**Published:** 2026-01-26

**Authors:** Eun-Soo Lee, Yoon-Seok Jang, Jae-Il Han, Tae-Rin Kim, Jae-Hwan Kim, Jae-Eun Hyun

**Affiliations:** 1 Department of Veterinary Internal Medicine, College of Veterinary Medicine, Konkuk University, Seoul, Republic of Korea; 2 Department of Veterinary Medical Imaging, College of Veterinary Medicine, Konkuk University, Seoul, Republic of Korea

**Keywords:** Budd–Chiari-like syndrome, feline, polycystic kidney disease, polycystic liver disease, whole-genome sequencing

## Abstract

A 9-year-old spayed female Persian Chinchilla cat presented with progressive lethargy and acute right hindlimb pain and lameness. Diagnostic imaging revealed diffuse renal and hepatic cysts, resulting in marked hepatomegaly. Computed tomography (CT) further identified a localized narrowing of the intrahepatic caudal vena cava (CVC), likely due to extrinsic compression by the enlarged liver. Laboratory tests revealed moderate anemia, leukocytosis, hypoalbuminemia, and a hypercoagulable state with markedly elevated serum amyloid A levels. Based on these findings, the cat was diagnosed with polycystic kidney and liver disease (PKD/PLD), complicated by Budd–Chiari-like syndrome (BCLS), a rare hemodynamic disorder in felines. Despite supportive care, the patient succumbed to renal failure within 7 weeks. Whole-genome sequencing identified a heterozygous known pathogenic non-sense mutation in *PKD1* (XM_023247051.2:c.9864C > A), a novel frameshift mutation in *GANAB*, and multiple missense variants in *PKD1* and *PKHD1*. To the best of our knowledge, this is the first reported case of feline BCLS secondary to PLD-induced CVC compression. These findings underscore the importance of considering vascular complications in advanced PKD/PLD and suggest that multigenic variation may contribute to disease severity and clinical variability.

## 1 Introduction

Polycystic kidney disease (PKD) is a common inherited disorder in cats, characterized by the formation of multiple renal cysts of varying sizes within the renal parenchyma. This condition is most prevalent in Persian and Persian-related breeds, such as Himalayan, Exotic Shorthair, and British Shorthair cats, with a reported prevalence of approximately 30–50% (1, 2). PKD is frequently accompanied by polycystic liver disease (PLD), and their concurrent occurrence in cats is well-documented (3, 4). In patients with PKD, renal cysts gradually replace normal renal tissue, leading to progressive kidney dysfunction and ultimately chronic kidney disease (CKD). Clinical signs depend on disease stage and may include polyuria, polydipsia, decreased appetite, vomiting, weight loss, and lethargy, all of which are characteristic symptoms of CKD (1). While PLD is typically asymptomatic, severe cases can result in hepatomegaly, abdominal distension, or, less commonly, hepatic dysfunction (3, 5).

These disorders generally progress slowly and are often diagnosed incidentally during imaging. Abdominal ultrasonography remains the most commonly used non-invasive diagnostic tool (6); however, computed tomography (CT) offers superior anatomical detail, allowing for better assessment of cyst size, distribution, and impact on surrounding structures (7). Feline PKD follows an autosomal dominant inheritance pattern, with mutations in *PKD1* recognized as the principal cause (8). Consequently, genetic testing for *PKD1* serves not only for early detection and risk assessment but also for complementing imaging in confirming the diagnosis of feline PKD (9). In contrast, the genetic basis of feline PLD remains largely undefined (5). Current management strategies for PKD and PLD are mainly supportive, aiming to maintain organ function, relieve clinical symptoms, and prevent complications. Targeted therapies are currently unavailable (1), highlighting the importance of early diagnosis, regular monitoring, genetic screening, and responsible breeding practices to mitigate disease progression.

Despite the relatively common concurrent presentation of PKD and PLD in cats, no reports to date have documented their association with Budd–Chiari-like syndrome (BCLS), a rare hemodynamic complication. This case report aimed to describe the clinical features, imaging findings, and genetic characteristics, identified through whole-genome sequencing (WGS), in a cat exhibiting this rare and complex disease presentation.

## 2 Case description

### 2.1 Case presentation and diagnostic investigation

A 9-year-old spayed female Persian Chinchilla cat was referred for evaluation due to the acute onset of right hindlimb pain, lameness, and progressive lethargy. Clinical signs had begun 5 days prior, with a marked decline in activity since onset. According to the medical history, intermittent vomiting had started approximately 7 weeks earlier, gradually increasing in frequency and persisting despite symptomatic treatment. On physical examination, the cat weighed 2.93 kg and had a body condition score of 3/9. In addition to pronounced abdominal distension (Supplementary Figure 1A), there was swelling extending from the proximal to distal aspect of the right hindlimb (Supplementary Figure 1B), accompanied by significant pain on palpation. A complete blood count revealed moderate anemia (hematocrit: 19.9%; reference interval [RI]: 30.3–52.3%), leukocytosis (31.99 × 10^3^/μL; RI, 2.87–17.02 × 10^3^/μL) with neutrophilia (25.02 × 10^3^/μL; RI, 1.48–10.29 × 10^3^/μL), and monocytosis (1.45 × 10^3^/μL; RI, 0.05–0.67 × 10^3^/μL). Thrombocytosis (772 × 10^3^/μL; RI, 151–600 × 10^3^/μL) was also noted. Serum biochemistry revealed mild hypoalbuminemia (2.1 g/dL; RI, 2.3–3.9 g/dL), with hepatic and renal parameters within reference intervals. Serum amyloid A (SAA) was markedly elevated (>500 mg/L; RI, 0–10 mg/L), although pancreatic lipase immunoreactivity and serum electrolyte concentrations remained within reference ranges. Thromboelastography revealed a hypercoagulable state. Urinalysis indicated decreased urine concentrating ability, with a urine-specific gravity of 1.024 (RI > 1.035) and proteinuria (urine protein-to-creatinine ratio: 0.91; RI < 0.2).

**Figure 1:**
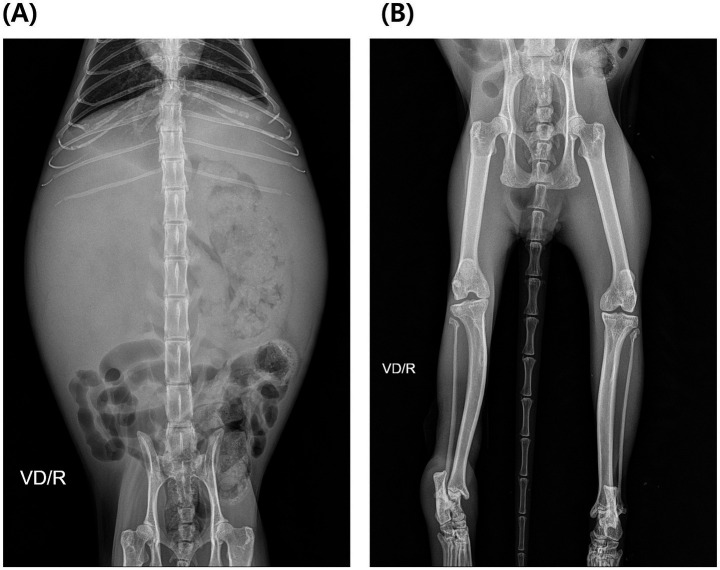
Radiographic examination findings: **(A)** decreased peritoneal serosal detail, displacement of the gastrointestinal tract, and poorly defined liver margin; **(B)** right hindlimb muscle atrophy and distal limb soft tissue swelling.

**Figure 2:**
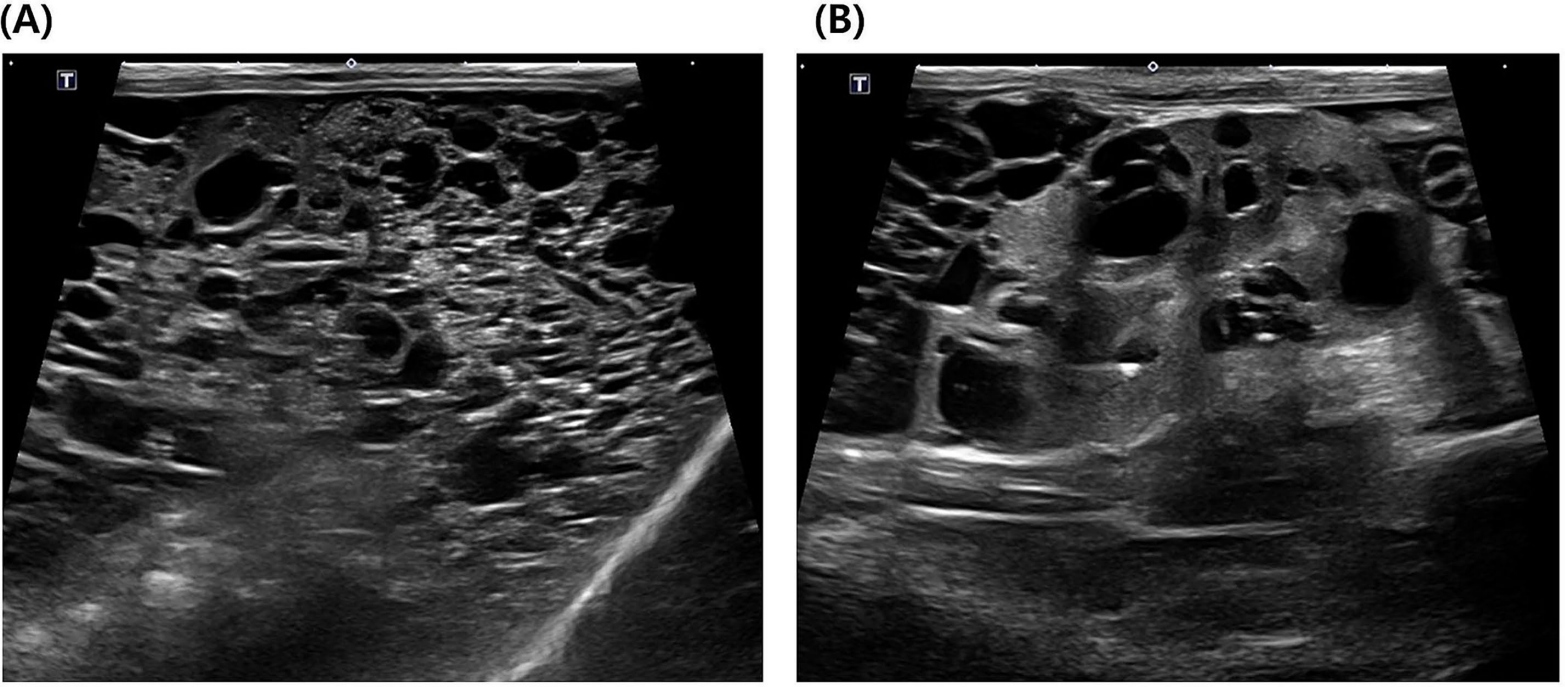
Abdominal ultrasound of the liver and kidney. **(A)** Multiple cystic lesions of variable sizes diffusely involving the entire liver parenchyma. **(B)** Cystic lesions in the kidney.

**Figure 3:**
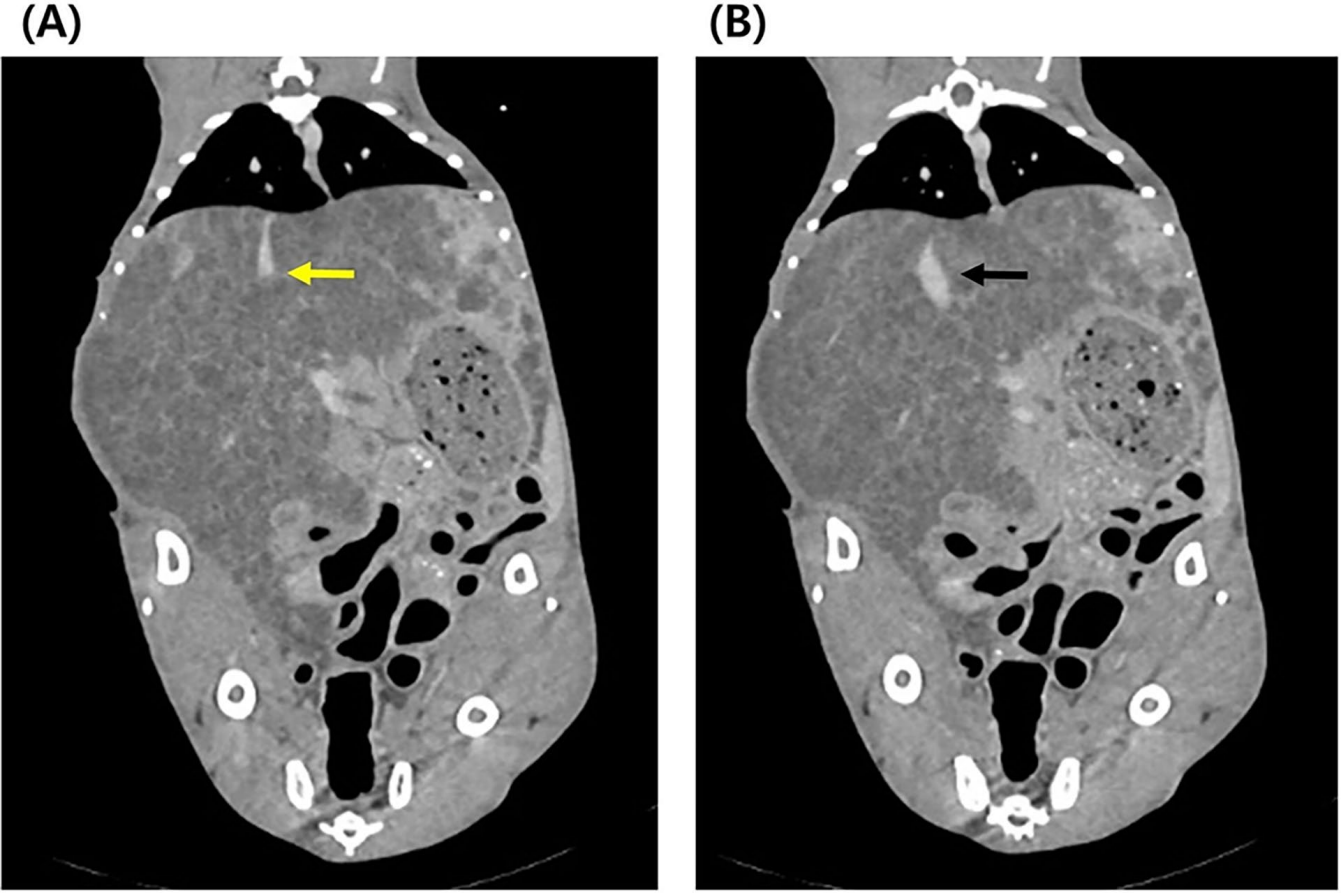
Abdominal computed tomography of the cat. **(A)** Compression of the caudal vena cava (yellow arrow) extending into the liver due to multiple cystic lesions. **(B)** Normal caudal vena cava (black arrow).

Radiographic examination revealed decreased peritoneal serosal detail and caudodorsal displacement of the gastrointestinal tract, with a poorly defined liver margin (Figure 1A). Right hindlimb muscle atrophy and distal limb soft tissue swelling were also observed (Figure 1B). Abdominal ultrasonography identified multiple variably sized cysts diffusely involving all hepatic lobes, accompanied by marked hepatomegaly occupying the majority of the abdominal cavity (Figure 2A). Cyst sizes ranged from <2 mm to 1.5 cm, with several exceeding 1 cm; due to their abundance, a total cyst count was deemed clinically irrelevant. Similar cystic lesions were present in both kidneys (Figure 2B), with the majority measuring 5–8 mm and some reaching 1.5–2.0 cm. The right kidney (44.7 mm) appeared slightly larger than the left (38.3 mm), with larger cysts, although the number of cysts was similar. Renal parenchyma appeared normal in echogenicity. Mild ascites was noted, without sonographic evidence of thrombosis. Echocardiography was unremarkable. CT confirmed severe hepatomegaly and numerous cystic lesions in the liver and kidneys, with residual normal parenchyma observed only in the left lateral, left medial, quadrate, and right medial lobes—all of which indicated normal contrast enhancement. Organ displacement and ascites were consistent with ultrasonographic findings. CT also revealed compression of the intrahepatic portion of the caudal vena cava (CVC), likely secondary to extensive hepatic cysts (Figure 3). At the T9–10 and T13–L1 levels, the CVC diameter was focally reduced from approximately 6.7 mm and 6.1 mm to 1.3 mm before returning to near-normal diameters of 5.9 mm and 5.6 mm, respectively.

Based on the imaging findings, this case was diagnosed as PLD/PKD considering the breed’s predisposition, laboratory results, and imaging evidence. The clinical signs, particularly right hindlimb swelling and pain, were attributed to venous congestion resulting from CVC compression secondary to marked hepatomegaly. Following the exclusion of other potential causes of hepatic venous outflow obstruction, the condition was considered BCLS secondary to PLD. Treatment was administered to alleviate clinical symptoms. A fentanyl patch (6 mcg/h) was administered for pain management, and antithrombotic agents (clopidogrel: 18.75 mg/cat q24h and rivaroxaban: 2.5 mg/cat q24h) were used to improve blood flow. Antibiotics (amoxicillin/clavulanic acid: 12.5 mg/kg q12h and metronidazole: 10 mg/kg q12h) were prescribed to prevent secondary infections. A gastric protectant (omeprazole: 1 mg/kg q12h), an antiemetic (maropitant: 1 mg/kg q24h), and an appetite stimulant (mirtazapine: 1.88 mg/cat q24h) were prescribed to manage gastrointestinal symptoms. Temporary pain relief was achieved following treatment; however, clinical signs rapidly progressed, and the patient succumbed to renal failure within 7 weeks of diagnosis. An additional genetic analysis was performed using WGS to investigate the underlying causes of the cat’s PKD and PLD.

### 2.2 Genetic analysis

DNA extraction and WGS were performed as follows. Genomic DNA was extracted from whole blood samples using the Maxwell^®^ 16 Blood Plus LEV DNA Purification Kit (Promega, United States) following the manufacturer’s instructions. Briefly, 300 μL of whole blood was mixed with 30 μL of proteinase K solution and 300 μL of lysis buffer and incubated at 56 °C for 20 min to ensure complete cell lysis. Subsequently, lysed samples were loaded into Maxwell^®^ 16 LEV cartridges and processed using the Maxwell^®^ 16 MDx Instrument. DNA was eluted in 120 μL of nuclease-free water and stored at −20 °C until further use. WGS library preparation and sequencing were performed using the Illumina DNA Prep Kit (Illumina, Inc., San Diego, CA, United States) according to the manufacturer’s instructions. Briefly, 100 ng of genomic DNA was fragmented and tagged with adapters by tagmentation, followed by polymerase chain reaction (PCR) amplification using Nextera indexed primers. The libraries were quantified using KAPA Library Quantification Kits (KAPA Biosystems, #KK4854) and assessed for quality using the D5000 ScreenTape (Agilent Technologies, Waldbronn, Germany). Indexed libraries were sequenced on an Illumina NovaSeqX platform (Illumina, Inc., San Diego, CA, United States) with paired-end 151-bp reads to achieve an average depth of 30 × coverage at Macrogen Incorporated.

For variant analysis, the obtained fastq files were aligned with the *Felis catus* reference genome (Felis_catus_9.0) using BWA-MEM (v0.7.17). Mutation analysis was performed on seven genes previously identified as causative for PKD in humans: *PKD1*, *PKD2*, *DNAJB11*, *GANAB*, and *LRP5* (associated with autosomal dominant polycystic kidney disease, ADPKD), as well as *PKHD1* and *DZIP1L* (associated with autosomal recessive polycystic kidney disease, ARPKD) (10, 11). Variant annotation and classification to identify the types and predicted impacts of the detected variants were performed using SnpEff (v4.3t) with the Ensembl *Felis catus* gene annotation corresponding to the Felis_catus_9.0 reference genome (Ensembl release 104). The Ensembl Variant Effect Predictor (v113.0) was additionally used to obtain Sorting Intolerant From Tolerant (SIFT) scores for missense variants, providing *in silico* predictions of potential functional relevance. The raw whole-genome sequencing data generated in this case have been deposited in the European Nucleotide Archive under the study accession number PRJEB105720 and the run accession number ERR16054094.

WGS identified a heterozygous known pathogenic non-sense mutation in *PKD1* (XM_023247051.2:c.9864C > A; chrE3:g.42858112C > A), which has been established as a causative variant for feline PKD. Further, a novel frameshift mutation in *GANAB* (chrD1:g.108403639delC) was detected, along with 20 missense mutations in *PKD1* and 17 in *PKHD1*. Functional predictions for the identified missense variants were obtained using the Ensembl Variant Effect Predictor, including SIFT scores. A comprehensive list of all non-synonymous variants identified in *PKD1*, *PKHD1*, and *GANAB*, annotated according to the Human Genome Variation Society nomenclature, is provided in Supplementary Table 1.

## 3 Discussion

In this case report, we describe the clinical presentation, diagnostic features, and prognosis of a Persian Chinchilla cat diagnosed with concurrent PKD and PLD. PKD is one of the most common genetic disorders in cats, and several epidemiological studies have shown that >90% of cats with PKD are Persian breeds (12, 13). In addition to the kidney, PKD patients may present with cysts in various organs, including the liver and pancreas, although hepatic cysts are the most common. The reported prevalence of concurrent renal and hepatic cysts varies across studies; one found that 12.6% of cats with PKD had coexisting hepatic cysts. For instance, the prevalence of PLD in Persian cats has been reported as high as 31% (4). The primary etiology of feline PKD is an autosomal dominant mutation in *PKD1*. This mutation leads to a functional defect in polycystin-1, resulting in impaired function of primary cilia in renal epithelial cells and subsequent cyst formation (12). Although the pathogenesis of PLD in cats is not yet fully elucidated, it is hypothesized that the same *PKD1* mutation may disrupt polycystin-1 function in hepatic tissue, thereby promoting cyst development within the liver parenchyma (3).

This patient presented with non-specific yet progressive clinical signs, including abdominal distension, lethargy, weight loss, and vomiting. Additionally, acute-onset swelling and pain in the right hindlimb were noted. Typical clinical manifestations of PKD and PLD in cats include polyuria, polydipsia, vomiting, weight loss, and abdominal enlargement—largely reflecting systemic effects of renal or hepatic dysfunction (1, 5). However, hindlimb swelling and pain are not typical features of either PKD or PLD alone and were instead presumed to result from vascular obstruction and perfusion impairment, likely secondary to BCLS. In Budd–Chiari syndrome (BCS), liver congestion, ascites, and hepatomegaly are due to occlusion of the hepatic vein or inferior vena cava, with various underlying causes in humans (14). In cats, BCLS has rarely been reported, with previously documented cases involving neoplasia or membranous stenosis as the underlying causes (15–19). In human patients with PLD, hepatic cysts have occasionally been implicated in the compression of hepatic veins leading to BCS (20, 21). However, to date, there are no reports of BCLS in cats secondary to PLD. Lower extremity edema is a recognized clinical feature in human BCS cases involving inferior vena cava obstruction (22). In the present case, the development of hindlimb edema and pain may reflect a similar pathophysiological mechanism, likely due to CVC compression.

In this case, abdominal ultrasound revealed >4 cysts, ranging from 5 mm to 2 cm in diameter, diffusely distributed throughout the parenchyma of both kidneys. Multiple hepatic cysts, measuring 2 mm to 1.5 cm in diameter, were also identified throughout the liver parenchyma, in a pattern similar to that of the kidneys. These imaging findings are consistent with previously established ultrasonographic diagnostic criteria for feline PKD (23). Importantly, CT identified a focal narrowing of the intrahepatic portion of the CVC, which was not apparent on ultrasound. This vascular compression was attributed to adjacent hepatic cysts. The marked reduction in CVC diameter, along with corresponding clinical signs of BCLS, strongly supports a hemodynamic consequence of this structural compression. Previous CT reports of BCLS in cats have primarily described CVC obstruction secondary to neoplasia or congenital membranous stenosis (15–17). While this case also showed significant CVC narrowing, unlike prior reports, BCLS here resulted from multiple liver cysts due to PLD compressing blood vessels. This case is the first clinically confirmed case of BCLS caused by PLD in a cat, suggesting that rare complications such as BCLS can also occur in cats with PLD, with important clinical implications.

At the time of diagnosis, the patient presented with moderate anemia, leukocytosis, and hypoalbuminemia, while liver and renal serum chemistry values were within normal limits. These findings indicated that the initial clinical signs were not attributable to overt hepatic or renal failure associated with PKD or PLD. However, as the disease progressed, elevations in blood urea nitrogen, creatinine, and phosphorus levels were noted, accompanied by worsening clinical symptoms. The patient ultimately succumbed to renal failure. Notably, a marked elevation of feline SAA and a hypercoagulable state were observed at initial presentation—an unusual finding considering that PKD typically follows a non-inflammatory chronic course. Despite proteinuria, renal biochemical parameters remained within reference intervals at that time, making an acute kidney injury unlikely. Taken together, the hematologic and biochemical abnormalities observed at initial presentation were considered to be more consistent with secondary effects related to PLD-associated mechanical processes rather than overt organ failure directly attributable to PKD or PLD itself. Feline PKD is generally characterized by a slowly progressive course, and uncomplicated PLD is often an incidental finding without overt clinical signs (1). Therefore, the presence of leukocytosis, anemia, hypoalbuminemia, and a hypercoagulable state in this case is more plausibly explained by hemodynamic disturbances and systemic inflammation resulting from severe hepatomegaly with compression of adjacent organs and blood vessels, including the caudal vena cava, or by inflammatory responses triggered by hepatic cyst rupture.

Genetic analysis revealed a heterozygous known pathogenic non-sense mutation in *PKD1*, which has been widely established as a causative variant for feline PKD, along with a novel frameshift mutation in *GANAB* and multiple missense variants in *PKHD1* and *PKD1*. Feline PKD has traditionally been associated with a specific *PKD1* mutation, c.10063C > A in exon 29 (12). However, recent cases without identified mutations have indicated the possibility of other mutations in candidate genes such as *PKD2*, *PKHD1*, *IFT80*, *ANKS6*, and *RPGRIP1L* (10, 24, 25). In humans, mutations in genes such as *PKD1*, *PKD2*, *GANAB*, *PKHD1*, *PRKCSH*, and *SEC63* have been reported to cause ADPKD, ARPKD, and autosomal dominant polycystic liver disease (ADPLD), and combinations of these genes are known to affect disease phenotype and severity (11, 26). Given the clinical and genetic similarities between feline and human PKD, there is likely genetic heterogeneity in cats, suggesting that multiple genetic variants may modulate the clinical manifestations of the disease (4, 25). *GANAB* mutations have been associated with hepatic cysts in humans and are believed to impair glucosidase II subunit *α*, leading to defective polycystin-1 maturation and subsequent cystogenesis in the liver and kidneys (27). In addition, other genetic mutations, such as *GANAB*, can influence the clinical diversity and disease progression of PKD, and even with the same *PKD1* mutation, the clinical presentation and prognosis can differ depending on the presence or absence of additional mutations (28, 29). In this case, a frameshift mutation in *GANAB* was identified in addition to the pathogenic *PKD1* mutation. Although the possibility that this *GANAB* variant may have influenced the clinical phenotype cannot be excluded, this observation is based on a single case, and any functional or clinical interpretation requires validation in larger cohorts of cats with well-defined phenotypes. In addition, several missense variants were identified in *PKHD1* and *PKD1*; however, their functional and clinical significance remains uncertain due to the lack of complementary population and functional data. Although *in silico* prediction tools, including SIFT, suggested possible effects for some variants, two *PKD1* variants (chrE3:g.42844184C > A and chrE3:g.42853493A > G) have been previously observed in phenotypically normal cats (8); thus, their pathogenicity remains uncertain and must be interpreted with caution. As this analysis was based on a single individual, the clinical significance of these polygenic variants cannot be generalized. Further studies involving larger sample sizes are warranted to clarify the roles of multigenic variation and identify genes associated specifically with feline PLD.

Treatment for feline PKD and PLD is largely supportive, focusing on preserving renal and hepatic function, alleviating clinical signs, and preventing complications (1). Interventions such as cyst drainage or sclerotherapy are rarely feasible due to the diffuse distribution and fragile nature of cysts (2, 30). In this case, percutaneous drainage or surgical excision was not pursued due to the owner’s refusal. Accordingly, conservative management was pursued to alleviate secondary pathophysiological consequences of the disease, including venous congestion-associated pain, hypercoagulability, systemic inflammation, and gastrointestinal signs. Although transient symptomatic improvement was observed, the limited overall clinical response was considered to reflect the advanced stage and multifactorial nature of the condition rather than inadequate therapeutic intervention. In humans, BCS has a well-established stepwise treatment strategy, including medical treatment, vascular intervention, transjugular intrahepatic portosystemic shunt, and liver transplantation, depending on its cause, symptom severity, and liver function status (22). In contrast, BCLS in cats is exceedingly rare, so no standardized treatment protocols have been established. In reported cases, either supportive care or treatment targeting the underlying cause when identifiable was provided (15–19). Thus, there is a need to develop further evidence-based treatment strategies and standardized guidelines in veterinary medicine. Prognosis in feline PKD and PLD is highly variable. One study reported a median survival of 12.7 years, with survival rates sharply declining after 6–7 years (31). In this case, the patient initially presented with clinical signs attributed to BCLS but progressed to CKD, dying 7 weeks after diagnosis. In humans, multi-organ polycystic disease is similarly associated with increased severity, complication risk, and poor prognosis (32). The rapid clinical deterioration and poor prognosis in this case may also have been due to complex organ involvement. However, the clinical course and prognostic patterns in cats with concurrent PKD and PLD are poorly characterized, underscoring the need for future research to clarify the natural history, risk factors, and outcomes associated with PKD/PLD.

In summary, this case illustrates the need for clinicians to be aware of possible rare complications such as BCLS in feline patients diagnosed with PLD. Further, as multiple genetic variants may influence the phenotypic expression and severity of PKD and PLD, there is a need for future in-depth studies to better understand the genetic basis and clinical heterogeneity of feline PKD and PLD, as well as to develop more effective treatment strategies for affected cats.

## Data Availability

The raw whole-genome sequencing data generated in this study have been deposited in the European Nucleotide Archive under the study accession number PRJEB105720 and the run accession number ERR16054094. The data are available at https://www.ebi.ac.uk/ena/browser/view/PRJEB105720.
