# Supplementary figures and images for: Case Report: Budd–Chiari-like syndrome in a cat with polycystic kidney and liver disease
Source: Front Vet Sci. 2026 Jan 26;12:1701832. doi: 10.3389/fvets.2025.1701832 (PMC12884389; doi:10.3389/fvets.2025.1701832)

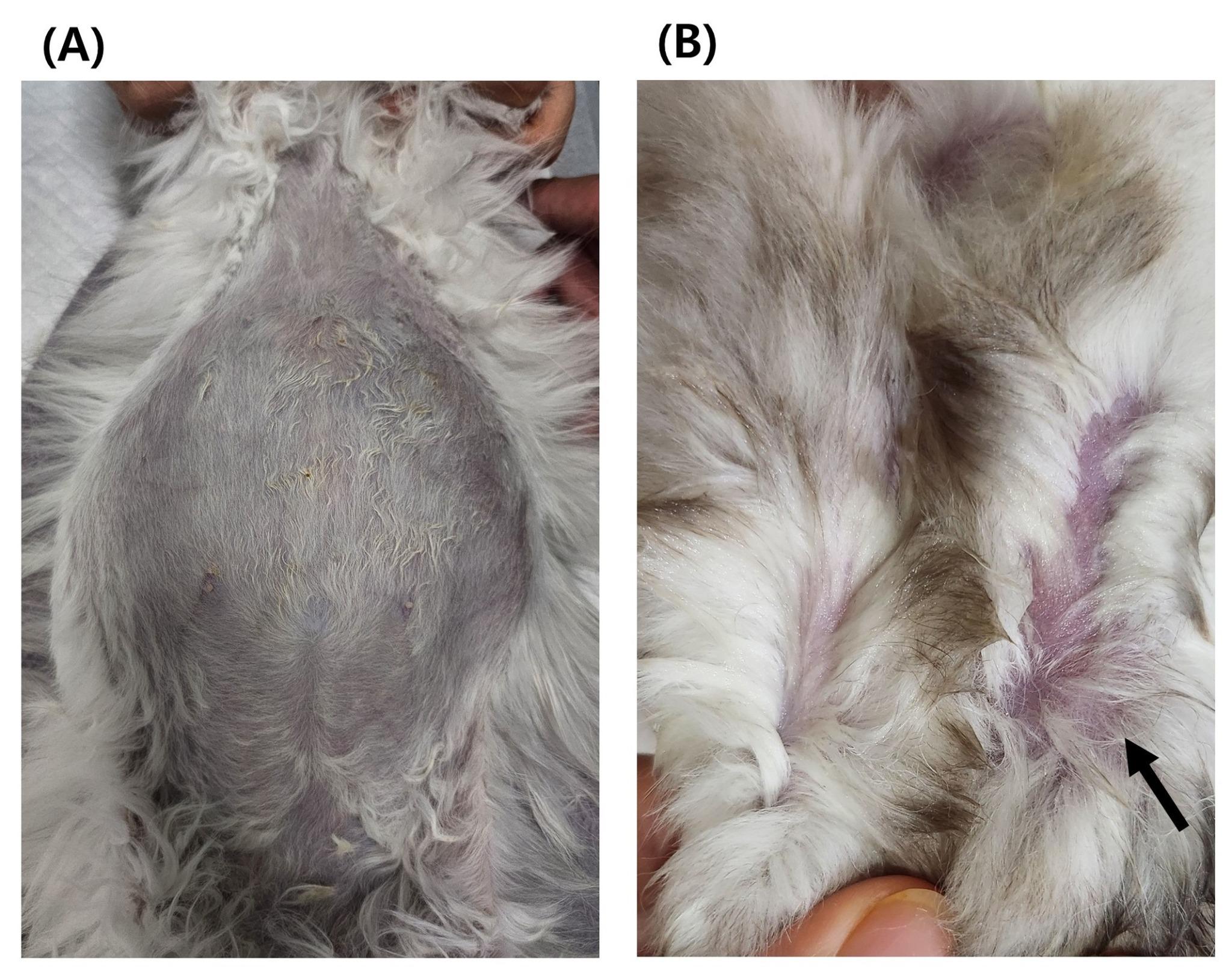

Supplement: Supplementary file 1 [file Image_1.jpeg]
