# Supplementary material for: Case Report: Budd–Chiari-like syndrome in a cat with polycystic kidney and liver disease
Source: Front Vet Sci. 2026 Jan 26;12:1701832. doi: 10.3389/fvets.2025.1701832 (PMC12884389; doi:10.3389/fvets.2025.1701832)
Supplement: Supplementary file 2 [file Table_1.docx]

Supplementary Material

**Supplementary Figure 1.** Physical examination findings. (A) Abdominal distention (B) swelling in the right hindlimb (arrow).

**Supplementary Table 1.** A total of 39 nonsynonymous variants of *PKD1*, *PKHD1*, and *GANAB* identified by whole-genome sequencing.

| Gene | Genomic coordinate | Reference accession | HGVS-c | HGVS-p | Variant type | Impact | Zygosity | OMIA variant ID | SIFT* |
| --- | --- | --- | --- | --- | --- | --- | --- | --- | --- |
| *PKHD1* | chrB2:g.52046775C>T | XM_023240027.2 | XM_023240027.2:c.11533G>A | p.(Val3845Ile) | missense | MODERATE | het | NA | Deleterious (0) |
| *PKHD1* | chrB2:g.52149096C>T | XM_023240027.2 | XM_023240027.2:c.10011G>A | p.(Met3337Ile) | missense | MODERATE | hom | NA | Tolerated (0.29) |
| *PKHD1* | chrB2:g.52152625A>C | XM_023240027.2 | XM_023240027.2:c.9424T>G | p.(Cys3142Gly) | missense | MODERATE | hom | NA | Tolerated (0.65) |
| *PKHD1* | chrB2:g.52255481T>C | XM_023240027.2 | XM_023240027.2:c.7786A>G | p.(Thr2596Ala) | missense | MODERATE | het | NA | Deleterious (0) |
| *PKHD1* | chrB2:g.52269290T>C | XM_023240027.2 | XM_023240027.2:c.7657A>G | p.(Thr2553Ala) | missense | MODERATE | het | NA | Tolerated (1) |
| *PKHD1* | chrB2:g.52269349C>G | XM_023240027.2 | XM_023240027.2:c.7598G>C | p.(Arg2533Thr) | missense | MODERATE | het | NA | Deleterious (0.02) |
| *PKHD1* | chrB2:g.52272081G>A | XM_023240027.2 | XM_023240027.2:c.7390C>T | p.(Arg2464Trp) | missense | MODERATE | het | NA | Tolerated (1) |
| *PKHD1* | chrB2:g.52296892T>G | XM_023240027.2 | XM_023240027.2:c.6848A>C | p.(Tyr2283Ser) | missense | MODERATE | hom | NA | Tolerated (0.43) |
| *PKHD1* | chrB2:g.52418026C>A | XM_023240027.2 | XM_023240027.2:c.5242G>T | p.(Gly1748Cys) | missense | MODERATE | het | NA | Deleterious (0) |
| *PKHD1* | chrB2:g.52420757C>T | XM_023240027.2 | XM_023240027.2:c.4576G>A | p.(Val1526Ile) | missense | MODERATE | het | NA | Tolerated (0.21) |
| *PKHD1* | chrB2:g.52420952T>C | XM_023240027.2 | XM_023240027.2:c.4381A>G | p.(Thr1461Ala) | missense | MODERATE | het | NA | Deleterious (0.03) |
| *PKHD1* | chrB2:g.52421411C>T | XM_023240027.2 | XM_023240027.2:c.3922G>A | p.(Glu1308Lys) | missense | MODERATE | het | NA | Deleterious (0.04) |
| *PKHD1* | chrB2:g.52424290A>G | XM_023240027.2 | XM_023240027.2:c.3452T>C | p.(Val1151Ala) | missense | MODERATE | het | NA | Tolerated (0.07) |
| *PKHD1* | chrB2:g.52450417C>T | XM_023240027.2 | XM_023240027.2:c.2488G>A | p.(Val830Ile) | missense | MODERATE | het | NA | Tolerated (0.15) |
| *PKHD1* | chrB2:g.52458753T>G | XM_023240027.2 | XM_023240027.2:c.2033A>C | p.(Glu678Ala) | missense | MODERATE | hom | NA | Tolerated (1) |
| *PKHD1* | chrB2:g.52488628T>C | XM_023240027.2 | XM_023240027.2:c.365A>G | p.(Gln122Arg) | missense | MODERATE | hom | NA | Tolerated (0.51) |
| *PKHD1* | chrB2:g.52492657A>G | XM_023240027.2 | XM_023240027.2:c.136T>C | p.(Tyr46His) | missense | MODERATE | het | NA | Deleterious (0.02) |
| *GANAB* | chrD1:g.108403639GC>G | XM_023242922.2 | XM_023242922.2:c.2350delG | p.(Ala784fs) | frameshift | HIGH | hom | NA | ㅡ |
| *PKD1* | chrE3:g.42839944G>A | XM_023247051.2 | XM_023247051.2:c.634G>A | p.(Ala212Thr) | missense | MODERATE | het | NA | Tolerated (0.06) |
| *PKD1* | chrE3:g.42843634A>G | XM_023247051.2 | XM_023247051.2:c.2002A>G | p.(Thr668Ala) | missense | MODERATE | het | NA | Tolerated (0.8) |
| *PKD1* | chrE3:g.42844184C>A | XM_023247051.2 | XM_023247051.2:c.2118C>A | p.(Asp706Glu) | missense | MODERATE | het | NA | Deleterious (0.03) |
| *PKD1* | chrE3:g.42847359A>C | XM_023247051.2 | XM_023247051.2:c.3557A>C | p.(Glu1186Ala) | missense | MODERATE | het | NA | Tolerated (0.08) |
| *PKD1* | chrE3:g.42847502A>G | XM_023247051.2 | XM_023247051.2:c.3700A>G | p.(Thr1234Ala) | missense | MODERATE | hom | NA | Tolerated (0.17) |
| *PKD1* | chrE3:g.42847833C>T | XM_023247051.2 | XM_023247051.2:c.4031C>T | p.(Thr1344Met) | missense | MODERATE | het | NA | Tolerated (0.07) |
| *PKD1* | chrE3:g.42848285A>T | XM_023247051.2 | XM_023247051.2:c.4483A>T | p.(Thr1495Ser) | missense | MODERATE | het | NA | Tolerated (1) |
| *PKD1* | chrE3:g.42848369A>G | XM_023247051.2 | XM_023247051.2:c.4567A>G | p.(Ile1523Val) | missense | MODERATE | hom | NA | Tolerated (0.57) |
| *PKD1* | chrE3:g.42849461G>A | XM_023247051.2 | XM_023247051.2:c.5659G>A | p.(Ala1887Thr) | missense | MODERATE | het | NA | Tolerated (0.12) |
| *PKD1* | chrE3:g.42849762T>C | XM_023247051.2 | XM_023247051.2:c.5960T>C | p.(Met1987Thr) | missense | MODERATE | hom | NA | Tolerated (1) |
| *PKD1* | chrE3:g.42850889A>G | XM_023247051.2 | XM_023247051.2:c.6931A>G | p.(Thr2311Ala) | missense | MODERATE | het | NA | Tolerated (0.62) |
| *PKD1* | chrE3:g.42853046C>T | XM_023247051.2 | XM_023247051.2:c.7763C>T | p.(Thr2588Met) | missense | MODERATE | het | NA | Tolerated (0.12) |
| *PKD1* | chrE3:g.42853138A>G | XM_023247051.2 | XM_023247051.2:c.7855A>G | p.(Ile2619Val) | missense | MODERATE | het | NA | Tolerated (0.2) |
| *PKD1* | chrE3:g.42853493A>G | XM_023247051.2 | XM_023247051.2:c.7886A>G | p.(His2629Arg) | missense_variant&splice_region | MODERATE | het | NA | Deleterious (0.04) |
| *PKD1* | chrE3:g.42853544A>G | XM_023247051.2 | XM_023247051.2:c.7937A>G | p.(Gln2646Arg) | missense | MODERATE | het | NA | Tolerated (1) |
| *PKD1* | chrE3:g.42855067G>C | XM_023247051.2 | XM_023247051.2:c.8669G>C | p.(Gly2890Ala) | missense | MODERATE | hom | NA | Tolerated (1) |
| *PKD1* | chrE3:g.42858112C>A | XM_023247051.2 | XM_023247051.2:c.9864C>A | p.(Cys3288*) | nonsense | HIGH | het | 314 | ㅡ |
| *PKD1* | chrE3:g.42859959C>A | XM_023247051.2 | XM_023247051.2:c.10486C>A | p.(Leu3496Ile) | missense | MODERATE | het | NA | Tolerated (0.71) |
| *PKD1* | chrE3:g.42862845A>G | XM_023247051.2 | XM_023247051.2:c.10732A>G | p.(Ser3578Gly) | missense | MODERATE | hom | NA | Tolerated (1) |
| *PKD1* | chrE3:g.42864749C>T | XM_023247051.2 | XM_023247051.2:c.11368C>T | p.(His3790Tyr) | missense | MODERATE | het | NA | Tolerated (1) |
| *PKD1* | chrE3:g.42864765T>C | XM_023247051.2 | XM_023247051.2:c.11384T>C | p.(Met3795Thr) | missense | MODERATE | het | NA | Tolerated (1) |
| OMIA variant IDs are provided for known functional variants where available; NA indicates variants without an OMIA variant ID. | | | | | | | | | |
| *Mutations with SIFT scores at or below 0.05 are typically regarded as likely to be deleterious. | | | | | | | | | |

**
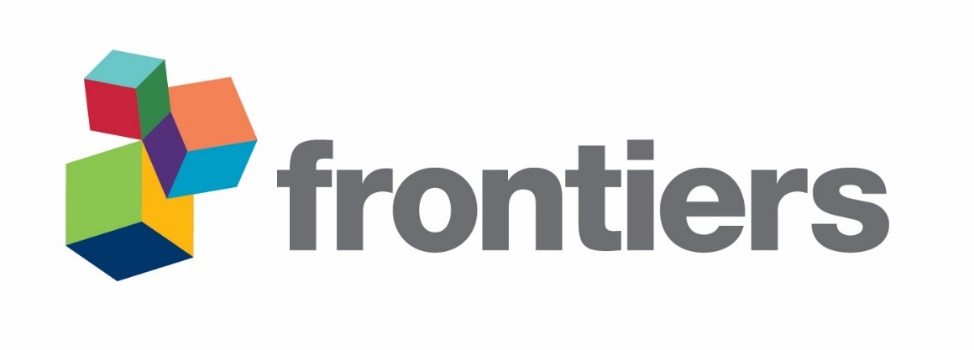
**
